# Dog Walking and the Social Impact of the COVID-19 Pandemic on Loneliness in Older Adults

**DOI:** 10.3390/ani11071852

**Published:** 2021-06-22

**Authors:** Dawn Carr, Erika Friedmann, Nancy R. Gee, Chelsea Gilchrist, Natalie Sachs-Ericsson, Lincy Koodaly

**Affiliations:** 1 Department of Sociology, Florida State University, Tallahassee, FL 32306, USA; 2 School of Nursing, University of Maryland, Baltimore, MD 21201, USA; friedmann@umaryland.edu (E.F.); lkoodaly@umaryland.edu (L.K.); 3 Center for Human-Animal Interaction, School of Medicine, Virginia Commonwealth University, Richmond, VA 23284, USA; Nancy.gee@vcuhealth.org; 4 Science Communications, IQ Solutions, Rockville, MD 20852, USA; cgilchrist@iqsolutions.com; 5 Department of Psychology, Florida State University, Tallahassee, FL 32306, USA; sachs@psy.fsu.edu

**Keywords:** human–animal interaction, aging, exercise, pet ownership

## Abstract

**Simple Summary:**

Growing evidence suggests that pets are beneficial to the health and wellbeing of older adults. These benefits have been more consistently shown among individuals navigating stressful situations and among those who have a strong attachment to their pet. Recent research suggests that older adults who walk their dogs experience fewer symptoms of loneliness than those who do not walk their dogs. The current study was designed to evaluate whether dog walking helps older adults facing significant social consequences related to the COVID-19 pandemic avoid increased feelings of loneliness. Our study shows that those who reported that COVID-19 had a significant impact on their social lives reported higher levels of loneliness, but if they walked their dog at least once a day, they did not become lonelier despite the social consequences of the pandemic. We conclude that dog walking could be a beneficial therapy in relation to loneliness for individuals who experience significant social setbacks. Further research is needed to determine why dog walking is beneficial, and whether individuals only obtain these benefits when walking their own dog, or if they can also experience benefits when walking with other people’s dogs.

**Abstract:**

Pet ownership can provide important companionship and facilitate social connections, which may be particularly important to socially isolated older adults. Given the significant deleterious impact of loneliness on health and wellbeing in later life, many predicted that public safety measures imposed during the COVID-19 pandemic would greatly increase loneliness, particularly among vulnerable populations like older adults. We investigated whether dog walking buffers loneliness in the context of stressors imposed by the COVID-19 pandemic. Longitudinal survey data were obtained from a Florida community-based sample of adults (*n* = 466) aged 60+ years old in September 2018 and October 2020. Using OLS regression models, we tested: a) the association between the social consequences of COVID-19 and changes in loneliness, and b) the buffering effect of dog walking on this relationship. The high social consequences of COVID-19 were related to increases in loneliness. Walking a dog daily buffered the relationship. These results suggest potential therapeutic effects of dog walking for the promotion of mental health in older adults, particularly in the context of stressful situations that accentuate risks for loneliness.

## 1. Introduction

The COVID-19 pandemic has had a profound effect on people of all ages, worldwide. However, older adults have been uniquely affected by the pandemic. The global COVID-19 mortality rates are elevated in adults over the age of 60, especially among those over 80, with those aged 80–89 experiencing more than quadruple the case fatality rate (RRR = 4.47, 95% CI = 4.1, 4.8), and those aged 90 and above have an even higher case fatality (RRR = 4.83, 95% CI = 4.4, 5.3) compared with those aged 60–69 years old [1].

The physical health risks related to the COVID-19 pandemic coincided with another public health problem—the growing proportion of U.S. older adults that indicate feeling lonely. Unfortunately, to reduce the spread of COVID-19, jurisdictions around the world determined that to protect the public health of all citizens and especially older adults, restrictions on social behaviors were needed. Older adults in particular were advised to eliminate interactions with others and shelter in place whenever possible [2]. Many community senior centers were closed. Senior living communities that normally offer many social and recreational activities isolated residents in their units and eliminated group activities. The impact of these quarantine and social distancing requirements may have been exacerbated by prevalent preexisting loneliness and social isolation in older adults. Studies of older adults in the U.S. [3] and Australia [4] confirmed that individuals over 60 years of age were particularly prone to loneliness during COVID-19 restrictions. At the beginning of the 2020 pandemic, older adults who had to cancel or postpone social activities experienced a 36% increase in odds of loneliness, and those who avoided close contact with others experienced a 41% increase in odds of loneliness [5].

Despite the mortality risks and restrictions on social behaviors related to COVID-19, not all older adults have experienced negative consequences from the pandemic [6]. Older adults with pets, in particular, may have maintained wellbeing despite restrictions on in-person interactions with others. Growing evidence suggests that pets, and dogs in particular, could play an important role in helping older adults avoid feelings of loneliness, particularly in the context of stressful life events [7], including social stressors like those imposed by the COVID-19 pandemic. An online survey of U.S. adults who teleworked from home during COVID-19 showed that individuals with dogs were likely to socialize with other people than individuals who did not own dogs [8]. The possibility that dogs might decrease the impact of social isolation for older adults was also supported by an online survey among a broad sample of Australians who lived alone during the COVID-19 lockdown. Dog ownership was associated with lower loneliness, even after accounting for cat ownership, mindfulness, anxiety, depression, and stress [9].

Growing evidence suggests that having a dog may be particularly beneficial when owners regularly walk them. Dog walking has been implicated as a means to increase opportunities for social interaction and improve psychological health for older adults [10]. Older adult dog owners who are socially isolated may be motivated to walk their dogs, offering several downstream positive effects likely to reduce loneliness such as increased physical activity, improved mental health, and increased opportunities for social interactions. Furthermore, long-term dog ownership has been associated with improvements in physical and social functioning for older adults who walk their dogs [11]. The current study examines the extent to which dog walking may have buffered the negative social consequences of COVID-19 on feelings of loneliness. Based on our results, we discuss the potential clinical benefits of dog walking for loneliness in the context of stressful life events.

### 1.1. Loneliness in Later Life

Loneliness is pervasive in the U.S. and around the world. In 2018, a national survey reported that 20% of Americans rarely or never felt close to people and 18% felt like they did not have people they could talk to [12]. These rates are alarming when considering the strong association between loneliness and adverse health outcomes in late life. Specifically, loneliness is associated with increased cognitive decline, poorer overall cognitive performance, and increased risk of depression [13,14,15,16,17]. Loneliness is also associated with a decline in mobility [18], increased risk of cardiovascular disease, and increased risk for early mortality [19,20,21,22].

Loneliness is related to perceptions of connectedness with others [23,24]. Maintaining high-quality social relationships with others is a key factor that prevents individuals from feelings of lonelines [25]. For instance, those who regularly engage in meaningful, productive activities with others experience fewer feelings of lonelines [15]. Alternatively, those whose social relationships become strained over time are more likely to become lonely [25]. The COVID-19 pandemic had a significant impact on social relationships. Given the disproportionate mortality risks of COVID-19 for older adults, physical distancing requirements were more important for older adults than those of younger ages. Adding to the impact of COVID-19, before the pandemic older adults were more likely than younger people to live alone and be unmarried, to experience the loss of a spouse to COVID-19 or other causes, and to maintain lower levels of social engagement, all factors that place this group at higher risk of loneliness than younger adults [26]. As a result, the social consequences of the pandemic, such as decreased sense of social connectedness with others, may be particularly likely to contribute to accelerated rates of loneliness in older adults.

### 1.2. Benefits of Pets

Pets have the potential to buffer the psychological health consequences of the COVID-19 pandemic, especially loneliness. Stress reduction is the most robust association related to human–animal interaction and older adults’ health and wellbeing [27]. The biopsychosocial model is an excellent framework to explain potential beneficial psychological associations related to pets in the context of COVID-19. The model emphasizes the interplay of the biological, psychological, and sociological factors defining one’s health. Any disturbances in any of these factors, such as stressors imposed by COVID-19, could impede health [28] including psychological health. Applying the biopsychosocial model, pet ownership is theorized to promote health by decreasing stress and stress responses, increasing social interaction and purpose in life, and providing an impetus for physical activity.

Pet ownership has been shown to buffer the impact of stressful life events [29]. Pets have also been shown to reduce feelings of social rejection in younger adult populations [30], and the presence of a friendly animal has been shown to be beneficial for reducing stress levels [31,32,33]. Numerous experimental studies show a reduction in stress biomarkers or anxiety when a friendly dog is present [27,34], and may reduce stress responses even more than the presence of other supportive people [35]. In studies of young adults and children, stress responses have been shown to decline when a companion animal is present [36,37,38,39].

Despite beneficial associations between pets and stress responses, the moderating influence of the presence of a companion animal on stress is not universal. In studies evaluating biological and psychological assessments of stress, results have been inconsistent. In one study, biomarker stress responses were not attenuated by the presence of an animal, but the presence of a dog moderated psychological stress response indicators [40]. In another study, the biomarker indicator of stress response was moderated by the presence of a dog, but the psychological measure was not [41]. Studies of animal interventions for older adults indicate that active interactions with dogs lead to increased social interaction [42,43] and physical activity [44], which could lead to additional beneficial effects, such as decreased loneliness. These effects, however, seem to be contingent with higher levels of engagement with dogs [44,45]. These behaviors suggest higher levels of pet attachment is linked to reduced loneliness [46].

Although pet ownership has the potential to moderate the relationship between social vulnerability and loneliness, evaluating these benefits can be challenging because loneliness is also associated with seeking out a pet [47]. That is, although pet ownership can lead to reduced loneliness [48,49,50,51], some individuals seek out pets because they feel lonely. Some studies show that pet owners report higher loneliness than non-owners [52], and have fewer friends than non-pet owners [53]. Consequently, to understand the potential benefits of pets for psychological health, it is important to account for social and emotional vulnerabilities.

### 1.3. Benefits of Dog Walking

Although pet ownership is generally associated with health benefits overall, dog walking may be particularly beneficial for promoting a sense of social connectedness and reduced loneliness. These benefits may be related to the extent to which dog walking promotes opportunities for social engagement with others, and increases in overall physical exercise which can indirectly lead to improved psychological health. Walking is the most adhered-to form of exercise for older adults [54]. Consequently, walking a dog has the potential to play an important role in supporting longer-term health and wellbeing benefits among older adults.

The preponderance of cross-sectional studies that examine the relationship of exercise to pet ownership demonstrates that older adult dog owners walk more than non-pet owners [55,56,57,58,59,60,61,62]. Walking a dog can also catalyze owners to interact with other people [63]. Focus groups with dog owners showed walking their dogs led to increased frequency of social interactions, especially with strangers [64]. The presence of a dog seems to act as an “ice breaker” by providing a neutral and safe opening for a conversation, and may change perceptions of strangers’ likeability [65]. No matter the reason that dog walking facilitates social interaction, it appears that dog walkers’ conversations with others during their walks are associated with decreased loneliness.

Even though there are documented benefits of dog walking, not all older adults may experience benefits from dog walking. Distinctive typologies of pet owners and non-owners show heterogeneous reasons for having pets, and the potential benefits of pets could differ based on these profiles [66]. Some older individuals may not experience improvements in wellbeing from dog walking because they are already thriving. Others may have challenges or characteristics that prevent them from being able to walk a dog. A variety of factors can influence if, when, and under what circumstances walking a dog may be beneficial [27,67]. For instance, in focus groups, some dog owners reported feeling anxiety about dog behaviors and social encounters with other people while they were walking their dogs [68]. Such concerns could lead people to avoid engaging with others while walking, decrease the length of time spent walking, or lead to poorer emotional responses in association with dog walking. In addition, walking dogs can lead to injuries to older owners. Dogs who pull during walks can cause rotator cuff injuries to their older owners and older owners who may have impaired balance or diminished vision may be particularly vulnerable and subject to injuries due to tripping or falling over their own dogs, other dogs, or objects on their walks [69]. Encounters with others’ animals during dog walks could also result in the transmission of infectious diseases and conflicts between a person’s dog and a strangers’ dog could result in injuries to owners and their dogs including bites and scratches [70,71]. Efforts to gain insights about causal associations between dog walking and psychological wellbeing require consideration of baseline characteristics and changes in wellbeing over time, not just evaluations of associations at a single point in time.

### 1.4. The Current Study

The current study is designed to evaluate the potential benefits of dog walking in buffering the social consequences of COVID-19 on loneliness. We consider the fact that dog walking may not be associated with reduced loneliness in all older adults. For instance, older adults who are already doing well (i.e., not lonely) and who maintain meaningful connections with others may not experience additional benefits from dog walking. However, walking a dog regularly could be uniquely protective for older adults who are socially isolated or socially vulnerable [29,47,72]. In the context of the social stressors imposed by COVID-19, dog walking could be particularly beneficial for those facing significant social setbacks. Specifically, guided by the literature reviewed above, this study is designed to test two hypotheses:

Specifically, we hypothesize:

**Hypothesis** **1** **(H1).** *Those who experience social consequences of COVID-19 will experience increases in overall loneliness.*

**Hypothesis** **2** **(H2).** *However, among individuals reporting significant social consequences of COVID-19, dog walking will buffer the effects of loneliness.*

## 2. Materials and Methods

### 2.1. Data and Sample

Data are drawn from a community-based sample of adults aged 60+. Under the initiative of the Institute for [Blinded for Review], older individuals in Florida were initially contacted (through mailings, advertisements, and social media) and agreed to join a registry for potential participation in aging-related research. The registering was voluntary, and individuals were not obligated to participate in a study. Registry participants were initially invited via e-mail to participate in the first wave of our survey, administered using Qualtrics in September 2018. A total of 906 individuals completed all questions in the baseline survey. In June 2020 and again in October 2020, we resurveyed these individuals. The current study is based on questions drawn from survey items collected at baseline (November 2018) and in October 2020 (Time 2), in which a total of 473 people (52% of the baseline) completed the survey. The Florida State University Institutional review board provided approval and oversight of our study. All individuals involved in the study provided informed consent prior to completion of each survey. Stata 14.2 was used for analysis of the survey data.

### 2.2. Measures

*Outcome Measure.* Loneliness was obtained at baseline and Time 2. Loneliness is a composite measure based on a modified version of the University of California, Los Angeles (UCLA) Loneliness Scale, and drawn from the Health and Retirement Study (HRS) Psychosocial and Lifestyle Questionnaire [73]. Individuals were asked to indicate the frequency with which they felt they: ref. [1] lack companionship; ref. [2] feel left out; and ref. [3] feel isolated from others. Responses include 1 = hardly ever or never, 2 = some of the time, and 3 = often. The score is based on the average response across all items. For ease of interpretation, the loneliness measures were standardized in the regression models. The outcome measure for the regression models was an autoregressive change score calculated as the standardized change in loneliness, controlling for baseline differences in loneliness.

Loneliness in our sample is similar to an age-matched national sample drawn from the Health and Retirement Study during a non-COVID-19 period. In 2014, HRS respondents aged 60 years and older had an average loneliness score of 1.455 (SD = 0.53) relative to our sample average at baseline in 2018 of 1.42 (SD = 0.54) (See Table 1). Our sample, on average, experienced minimal change in loneliness pre- to post-COVID-19 (0.037 units). The HRS sample, on average, experienced a very small increase in loneliness during a non-COVID-19 period between 2014 and 2016 (0.19 units). Relative to a national sample, these average scores and changes suggest that the average older adult in our sample had a similar level of loneliness as a typical older adult aged 60+ during a non-COVID-19 period, and on average, did not experience increased loneliness.

*Primary Independent Variables.* Our primary independent variables for this study include the Social Impact of COVID-19 and Frequency of Dog Walking. The Social Impact of COVID-19 was based on the question: “How much is the COVID-19 outbreak impacting your sense of social connection?” Responses are coded: 0 = Not at all, 1 = very little, 2 = some, 3 = much, 4 = very much. The Frequency of Dog Walking was collected only in the Time 2 survey based on the question: If you have a dog, how often do you walk your dog? Answers ranged from 0 = I don’t have a dog/I don’t walk my dog, 1 = less than once per week, 2 = 1–3 days per week, 3 = 4–7 days per week, 4 = once per day, to 5 = more than once per day.

*Control Measures.* We controlled for several factors, including measures related to other COVID-19 related exposures, other pet ownership, demographic factors, and social network related measures. Regarding other COVID-19 relative measures, first, we controlled for the Health Impact of COVID-19 based on the question: “How much of a threat is COVID-19 on your health or the health of your loved ones?” Second, we controlled for the Financial Impact of COVID-19 based on the question: “How much is the COVID-19 outbreak impacting your finances?” For these two questions, responses were coded: 0 = not at all, 1 = very little, 2 = some, 3 = much, 4 = very much. We also included a measure to account for the stressful societal events that were occurring in coordination with the pandemic that may have had an impact on loneliness and the overall sense of social connectedness. The Impact of Stressful Societal Events was measured at Time 2 based on the question: “In thinking about current events other than COVID-19, how much are each of the following contributing to your overall stress or worry over the last month?: ref. [1] the US presidential election, [2] racial injustice, [3] increases in white nationalism, [4] the economy, [5] lack of respect for the police, [6] the extent to which Americans are divided, and [7] the environment.” Response options ranged from 0 = none at all, 1 = a little, 2 = a moderate amount, 3 = a lot, and 4 = a great deal. This measure was calculated as the average response across all seven items.

Second, we controlled for pet ownership, including measures for Dog and Cat Ownership. Dog and cat ownership were assessed at baseline and at Time 2, resulting in four dichotomously measured variables, indicating whether an individual had at least one dog/cat at the time of each survey.

Third, we included several control measures related to demographic characteristics. Race was measured dichotomously as Minority (coded “1” for those non-White or Hispanic) versus non-Hispanic White (coded “0”). Educational attainment was measured: 1 = high school or less, 2 = some college, 3 = Bachelor’s Degree, 4 = Master’s Degree, and 5 = Doctoral Degree. Age was based on baseline survey age and included as a continuous measure ranging from 60–92 years. Gender was coded based on whether individuals identified as female (1 = female, 0 = male gender). Marriage was measured at baseline and at Time 2, and dichotomously coded based on whether individuals indicated being married or in a long-term partnership with another individual (1 = married/partnered, 0 = not married). Employment was measured at Time 2 based on whether an individual indicated working for pay (1 = employed, 0 = not employed). Self-rated health was based on one question collected at baseline, “Would you say your health is excellent, very good, good, fair, or poor?” and responses ranged from 1 (poor) to 5 (excellent).

Fourth, we included social network-related control measures. Our social network-related measures accounted for the quality of social relationships with friends, which may play a role in shaping loneliness [25,47,74]. We included two types of friendship quality measures drawn from the Health and Retirement Study Psychosocial and Lifestyle Questionnaire [73]. First, Social Support from Friends was measured at both Baseline and Time 2, and based on three questions assessing how respondents felt about friends they are close with (e.g., “How much do they really understand the way you feel about things?”). Participants responded on a 4-point Likert-type scale (1 = Not at all, 2 = A little, 3 = Some and 4 = A lot) (α = 0.89). For respondents who were missing more than one item, their score was coded as missing. Second, Social hassles from friends was measured at Baseline and Time 2 and included to account for “negative” social support that may influence loneliness. Social hassles were measured based on three questions assessing how respondents felt about friends they are close with (e.g., in relation to your friends “How often do they make too many demands on you?”). Participants responded on a 4-point Likert-type scale (1= Not at all, 2 = A little, 3 = Some and 4 = A lot) (α = 0.75). For respondents who were missing more than two items, their score was coded as missing. Individuals who indicated that they did not have any close friends did not respond to questions about social support from friends, so they were assigned a score of “0” for both perceived social support from friends and social hassles (*n* = 46). In addition to relationship quality, we also accounted for the potential effect of the death of a friend on overall loneliness. Friend Loss was measured at Time 2, based on whether the respondent indicated that, since March of 2020, they had experienced the death of a close friend (1 = friend loss, 0 = no friend loss).

### 2.3. Analytic Approach

To address our study hypotheses, we used individual OLS regression models to evaluate change in loneliness between September 2018 (pre-COVID-19 pandemic) and October 2020 (six months into the COVID-19 pandemic). We used robust standard errors to address heteroskedasticity. Listwise deletion was used to address missing data. Results are provided in Table 2. First, we evaluated the direct effects of the Social Impact of COVID-19, net of other negative consequences of COVID-19, on change in loneliness (Model 1). We further evaluated the Social Impact of COVID-19, controlling for [1] dog walking and having pets (Model 2), and [2] net of all other statistical controls (Model 3). Finally, we evaluated whether dog walking buffers the Social Impact of COVID-19 using interaction models (Model 4). Specifically, we interacted dog walking on the Social Impact of COVID-19 measure. To evaluate the association more thoroughly between these two measures, we calculated the marginal effects, predicting the average change in loneliness based on the amount of dog walking for those with the highest and those with the lowest scores on Social Impact of COVID-19. We calculated whether the amount of dog walking was significantly (i.e., *p* < 0.05) associated with a change in loneliness for each of these groups.

## 3. Results

### 3.1. Characteristics of Study Sample

Table 1 provides the overall characteristics of our community sample. Our sample reported a very similar level of loneliness at both time points, despite exposure to the COVID-19 pandemic. Overall, the average person in our sample reported a score of about 2.5 (range is 0–4) for the social impact of COVID-19, suggesting a substantial social impact on average. The sample also reported similarly high impacts on health (2.3) and in relation to societal events (2.0), but a fairly low impact on finances (0.7). The average amount of dog walking at Time 2 for our sample was 1.2, which is between once a week and 1–3 times per week. Approximately 42% of the sample had a dog at baseline and 40% had a dog at Time 2, and 30% had a cat at the baseline and 29% at Time 2.

### 3.2. OLS Regression Results

OLS regression models are provided in Table 2. Regarding our first hypothesis, models 1–3 show that the social impact of COVID-19 is significantly associated with increased loneliness. Specifically, each unit increase in the social impact of COVID-19 is associated with about one-fifth of a standard deviation increase in loneliness, even when controlling for pet exposures, other measures related to social relationships, and demographic factors.

**Figure 1:**
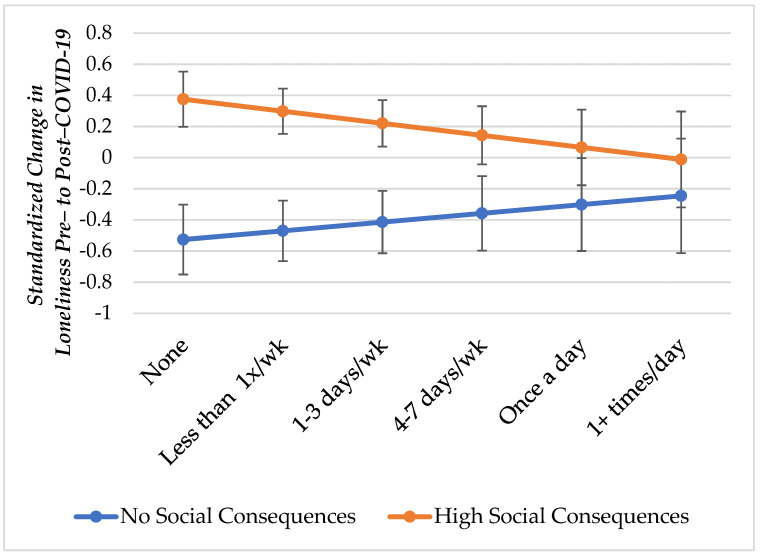
Buffering Effects of Dog Walking on the Social Consequences of COVID-19.

To address our second hypothesis, we used moderation tests to evaluate whether dog walking buffered the negative consequences of the social impact of COVID-19 on loneliness. Model 4 showed that the interaction between the social impact of COVID-19 and dog walking was statistically significant and negative, suggesting a salubrious effect of dog walking. However, to evaluate these findings more thoroughly, and to evaluate our hypotheses, we calculated marginal effects, predicting the average change in loneliness in association with dog walking for those with high and low social impact scores. Results are shown in Figure 1. Specifically, we found that for those who reported no social consequences associated with COVID-19, dog walking was not statistically associated with a change in loneliness. That is, those in this group experienced an improvement (i.e., a decrease) in loneliness regardless of how much they walked their dog. However, for those who reported the highest social consequences of COVID-19, dog walking was significantly associated with changes in loneliness. Those who did not walk their dog or did not have a dog reported about a 0.4 standard deviation increase in loneliness in association with COVID-19. However, those who regularly walked their dog (at least once per day) experienced no significant increase in loneliness, with similar loneliness as those who had no social consequences of COVID-19.

#### Sensitivity Analyses

We also conducted sensitivity analyses to evaluate whether other COVID-19 related stressors (i.e., financial impact of COVID, health impact of COVID, and Impact of Stressful Societal Events) might also be moderated by dog walking. Our evaluation showed that those reporting even high levels of COVID-19 related effects across the domains of finances, health, and other societal/non-COVID-19 related stressors did not experience a statistically significant increase in loneliness on average. Further, dog walking did not significantly moderate these effects. It is plausible that other consequences to health and wellbeing may have occurred in association with these other COVID-19 related stressors, which should be evaluated in future research studies.

## 4. Discussion

This study evaluated: (a) the association between the social consequences of COVID-19 and older adults’ loneliness, and (b) whether dog walking played a role in buffering the loneliness associated with the social consequences of COVID-19. Based on our community sample of older adults, our findings showed that those who experienced higher levels of social consequences of COVID-19 experienced greater increases in loneliness (supporting Hypothesis 1). However, walking a dog at least once a day off-set increases in loneliness among older adults who experienced significant social consequences related to the COVID-19 pandemic (supporting Hypothesis 2). Specifically, those with high levels of social consequences experienced significant increases in loneliness, but if they walked their dog at least once a day, they did not experience increases in loneliness.

Our study makes important and novel contributions to the current literature on pets and wellbeing in later life. First, previous studies have shown inconsistent results regarding the benefits of pet ownership. A common problem relates to the definition of pet ownership [67]. Pet ownership is frequently defined as the presence of a pet in the home, without consideration of the relationship between the pet and the owner. The focus of our study on dog walking provides a better understanding of how an owner relates to a pet. Stress reduction, a key factor shown to explain the benefits of pets to wellbeing, has been found to be related to the level of engagement with pets, and pet attachment [30]. Our findings show that dog walking is a particular type of pet engagement that seems to offer particular benefits to social wellbeing. The moderating influence of dog walking on the relationship between social consequences of COVID-19 and loneliness suggests that social aspects of dog walking may be more important than physical activity aspects when it comes to psychological wellbeing [75]. Further research is needed to evaluate mechanisms that explain these effects.

Second, the current study makes an important and nuanced contribution by showing that pet engagement, and specifically dog walking, could be more important for one subgroup of the population than for others. This is important because identifying groups for whom dog walking could be most beneficial is a prime goal for human–animal interaction research [27,67]. In a 2015 review of the literature relating pet ownership to loneliness, Gibey and Tani [76] emphasized the small samples and cross-sectional nature of most studies of pet ownership and loneliness. At that time most evidence came from studies with service animals, rather than pets. They suggested that the lack of studies showing a relationship was not the same as having studies that demonstrated no relationship between pet ownership and loneliness. Although our study is not a nationally representative study, our findings suggest that by focusing on those most affected by the social consequences of the pandemic, we observe the potential therapeutic benefits of pets. Promoting pet engagement, especially dog walking, in socially vulnerable older adults may provide significant benefits to wellbeing.

Despite our important findings, our study has several limitations that should be considered and addressed in future research. First, our study did not specifically evaluate how dog walking leads to decreased loneliness in socially vulnerable adults. It may be that the dog walking forestalls loneliness in this group because of the increased opportunity for socializing [77,78]. Even though owners were encouraged to maintain social distance during the pandemic, greetings from a distance or the across-the-street conversations may have served to ameliorate further feelings of loneliness. Seeing the same group of people walking on multiple occasions may have facilitated new conversations and friendships. It is also possible that the physical activity associated with dog walking and the follow-on benefits of that activity for various aspects of physical and mental health and wellbeing [27,55] are responsible for this effect. Dog walking also may have ameliorated loneliness in this group by strengthening the older adult’s relationship with their dog [68], potentially deepening an existing, or providing a much-needed, source of attachment and social support [30,75,79,80]. Previous research showed that dog owners in comparison with owners of other pets spent more time with their pets and felt that their pets were more important to them [29]. Thus, dogs more than other pets may provide their owners with companionship and an object of attachment that is uniquely beneficial to staving off loneliness.

In addition, the sample of participants in this study was drawn from a voluntary study, with data collected online. This is part of the reason that our sample was less diverse and more highly resourced than typical populations of older adults. Consequently, the benefits we observe may be conservative relative to a typical population of older adults. Individuals with more resources available to them may be less stressed overall by the pandemic indicating the possibility that our results under-represent the true potential for dog walking to ameliorate loneliness in older adults, particularly in association with stressful periods like the COVID-19 pandemic.

We were also unable to evaluate the possible influence of changes in dog walking patterns from prior to the pandemic because we did not measure dog walking in the two years prior to the pandemic. People likely had different pet-related routines prior to, and during, the pandemic. For example, there are indications that many people who did not own pets before the pandemic acquired pets during that period, although in supplementary analyses, we observed a very low proportion of individuals in our sample who became pet owners over our two-year study period. Pandemic-related lockdowns changed many routines due to the closing of senior centers, the elimination of many social and recreational activities, and sheltering in place. Although our data offers a unique insight into the role dogs play during a particularly universal stressful time like a pandemic, the results we found may also represent a temporary shift in behaviors that will return to pre-pandemic routines. For this reason, more research is needed to replicate or extend our findings.

Finally, we did not measure the emotional connection to pets. The emotional connection (e.g., feelings of attachment to a pet) between an owner and their dog could account for the dog walking effect. That is, people who regularly walk a dog may be those who are emotionally closer to their dogs, and that connection may explain the social benefits observed in this study. Attachment to a dog may even explain different motivations to stay physically engaged with a dog and thus support the needs of their pet. Future research needs to consider pet relationship as a factor in shaping the benefits of dog walking in association with stressful events.

Despite the limitations of our study, our findings provide promise for potential therapeutic benefits of pets in older adults. Our results suggest that it may be useful to support socially vulnerable older adults by facilitating dog walking, but this needs to be replicated by national studies while also assessing potential risks involved in dog walking activities for some older adults. Future research should evaluate whether benefits are contingent on walking one’s own dog, or if it might be possible to observe these benefits by walking a dog as a shelter volunteer or in an animal assisted therapy environment. However, dog walking is a relatively easy and inexpensive intervention, and given that older adults are more likely to adhere to walking than other forms of exercise, dog walking may offer a variety of benefits that older people can maintain for a long period of time.

## 5. Conclusions

This study adds to a growing base of literature showing the benefits of pets in the wellbeing of older adults. Our findings indicate that in the context of the COVID-19 pandemic, many older adults experienced significant setbacks regarding their social connection with others. These setbacks contributed to increases in loneliness in this population. However, among those who experienced these setbacks, regularly walking a dog was protective against significant increases in loneliness. If our findings are replicated in a national sample of older adults, these findings suggest potential clinical benefits for the psychological wellbeing of older adults who regularly engage in dog walking.

## Figures and Tables

**Table 1:** Characteristics of Study Sample (N = 466).

	Mean/Proportion	SD	Min	Max
Loneliness (Time 2)	1.387	0.540	1	3
Loneliness (Baseline)	1.424	0.542	1	3
Key Independent Measures				
Social Impact of COVID	2.480	1.105	0	4
Frequency of Dog Walking	1.207	1.958	0	5
Control Measures				
Financial Impact of COVID	0.736	0.934	0	4
Health Impact of COVID	2.304	1.046	0	4
Impact of Stressful Societal Events	2.006	0.704	0	4
Dog (Baseline)	41.6%			
Dog (Time 2)	39.5%			
Cat (Baseline)	29.8%			
Cat (Time 2)	28.8%			
Minority	8.0%			
Educational Attainment	3.300	1.047	1	5
Age	69.431	6.057	60	92
Female	66.0%			
Married (Baseline)	60.9%			
Married (Time 2)	61.3%			
Employed (Baseline)	28.5%			
Employed (Time 2)	21.8%			
Self-Rated Health (Baseline)	3.685	0.890	1	5
Social Support, Friends (Baseline)	3.056	0.992	0	4
Social Support, Friends (Time 2)	3.060	1.056	0	4
Social Hassles, Friends (Baseline)	1.275	0.514	0	4
Social Hassles, Friends (Time 2)	1.208	0.506	0	3.25
Friend Loss	19.5%			

**Table 2:** Evaluating the Buffering Effects of Dog Walking on the Social Consequences of COVID-19, Predicting Change in Loneliness Pre-COVID-19 relative to Six Months into COVID-19.

	Model 1	Model 2	Model 3	Model 4
	Beta (Robust SE)	Beta (Robust SE)	Beta (Robust SE)	Beta (Robust SE)
Loneliness (Baseline)—Standardized	0.585 ***	0.583 ***	0.522 ***	−0.482 ***
	(0.0500)	(0.0495)	(0.0539)	(0.0540)
Social Impact of COVID	0.192 ***	0.196 ***	0.187 ***	0.225 ***
	(0.0386)	(0.0379)	(0.0392)	(0.0448)
Frequency of Dog Walking		−0.0270	−0.0261	0.0561
		(0.0289)	(0.0279)	(0.0437)
Frequency of Dog Walking X Social Impact of COVID				−0.0334 *
Control Measures				(0.0152)
Financial Impact of COVID	−0.0408	−0.0445	−0.0585	−0.0653
	(0.0438)	(0.0436)	(0.0450)	(0.0442)
Health Impact of COVID	−0.00426	−0.0143	−0.0110	−0.0107
	(0.0393)	(0.0393)	(0.0389)	(0.0386)
Impact of Stressful Societal Events	−0.0514	−0.0555	−0.0538	−0.0625
	(0.0555)	(0.0551)	(0.0559)	(0.0560)
Dog (Baseline)		0.145	0.105	0.125
		(0.146)	(0.153)	(0.154)
Dog (Time 2)		−0.114	−0.105	−0.117
		(0.183)	(0.186)	(0.187)
Cat (Baseline)		0.138	0.153	0.153
		(0.130)	(0.117)	(0.116)
Cat (Time 2)		−0.0289	−0.0581	−0.0552
		(0.132)	(0.117)	(0.116)
Minority			−0.0390	−0.0276
			(0.141)	(0.142)
Educational Attainment			0.0480	0.0533
			(0.0346)	(0.0343)
Age			−0.00353	−0.00404
			(0.00662)	(0.00656)
Female			0.126	0.130
			(0.0821)	(0.0813)
Married (Baseline)			0.0241	0.0288
			(0.172)	(0.172)
Married (Time 2)			−0.227	−0.237
			(0.170)	(0.170)
Employed (Baseline)			−0.0656	−0.0724
			(0.103)	(0.104)
Employed (Time 2)			−0.0639	−0.0569
			(0.116)	(0.116)
Self-Rated Health			−0.0276	−0.0286
			(0.0459)	(0.0455)
Social Support, Friends (Baseline)			0.0446	0.0404
			(0.0544)	(0.0544)
Social Support, Friends (Time 2)			−0.187 **	−0.189 **
			(0.0579)	(0.0587)
Social Hassles, Friends (Baseline)			0.0539	0.0571
			(0.0821)	(0.0824)
Social Hassles, Friends (Time 2)			0.109	0.105
			(0.107)	(0.111)
Friend Loss			0.111	0.107
			(0.100)	(0.0994)
Constant	−0.333 *	−0.324 *	0.194	0.158
	(0.153)	(0.155)	(0.663)	(0.654)
R-squared	0.422	0.429	0.470	0.319

Note: N = 473; Robust standard errors in parentheses. Significance indicates: *** *p* < 0.001, ** *p* < 0.01, * *p* < 0.05. Model 1 includes only COVID-19 related measures. Model 2 adds in the pet-related measures. Model 3 adds in all control measures. Model 4 adds in the moderating effect of dog-walking on the social impact of COVID.

## Data Availability

These data are not pubicly available.
